# Prevalence, genotypes, and infection risk factors of psittacine beak and feather disease virus and budgerigar fledgling disease virus in captive birds in Hong Kong

**DOI:** 10.1007/s00705-024-06017-3

**Published:** 2024-04-05

**Authors:** Jackie Cheuk Kei Ko, Yannes Wai Yan Choi, Emily Shui Kei Poon, Nicole Wyre, Simon Yung Wa Sin

**Affiliations:** 1 School of Biological Sciences, The University of Hong Kong, Pok Fu Lam Road, Hong Kong, China; 2 Zodiac Pet & Exotic Hospital, 101A-103A Victoria Centre, 15 Watson Road, Fortress Hill, Hong Kong, China

## Abstract

Psittacine beak and feather disease virus (PBFDV) and budgerigar fledgling disease virus (BFDV) are significant avian pathogens that threaten both captive and wild birds, particularly parrots, which are common hosts. This study involved sampling and testing of 516 captive birds from households, pet shops, and an animal clinic in Hong Kong for PBFDV and BFDV. The results showed that PBFDV and BFDV were present in 7.17% and 0.58% of the samples, respectively. These rates were lower than those reported in most parts of Asia. Notably, the infection rates of PBFDV in pet shops were significantly higher compared to other sources, while no BFDV-positive samples were found in pet shops. Most of the positive samples came from parrots, but PBFDV was also detected in two non-parrot species, including Swinhoe’s white-eyes (*Zosterops simplex*), which had not been reported previously. The ability of PBFDV to infect both psittacine and passerine birds is concerning, especially in densely populated urban areas such as Hong Kong, where captive flocks come into close contact with wildlife. Phylogenetic analysis of the *Cap* and *Rep* genes of PBFDV revealed that the strains found in Hong Kong were closely related to those in Europe and other parts of Asia, including mainland China, Thailand, Taiwan, and Saudi Arabia. These findings indicate the presence of both viruses among captive birds in Hong Kong. We recommend implementing regular surveillance for both viruses and adopting measures to prevent contact between captive and wild birds, thereby reducing the transmission of introduced diseases to native species.

**Supplementary Information:**

The online version contains supplementary material available at 10.1007/s00705-024-06017-3.

## Introduction

Psittacine beak and feather disease virus (PBFDV) and budgerigar fledgling disease virus (BFDV) are highly lethal and contagious to various avian species, particularly psittacines, which include many threatened species [1, 2]. These two viruses are prevalent worldwide, affecting both captive and wild bird populations [3, 4]. The diseases caused by PBFDV and BFDV are not only a great concern for conservation efforts but also hold significant importance in the field of avian health and welfare.

Psittacine beak and feather disease (PBFD), caused by PBFDV, poses a significant threat to various endangered parrot species. For instance, Cape parrots (*Poicephalus robustus*) in South Africa [5, 6], Mauritius parakeets (*Alexandrinus eques*) in Mauritius [7], and swift parrots (*Lathamus discolor*) [8] and orange-bellied parrots (*Neophema chrysogaster*) in Australia [9] are among the species affected. PBFDV was initially documented in Australia during the 1970s [10] and has since spread globally through bird trade [11]. Since then, the virus has been reported in both wild and captive birds in over 40 countries [3]. PBFDV, a member of the family *Circoviridae*, has a small genome of approximately 2 kb, consisting of two major open reading frames (ORFs) encoding a replicase-associated protein (Rep) and a capsid protein (Cap) [11]. Birds with acute PBFD, typically fledglings, often die within a few days, while those with chronic infections may act as lifelong carriers [12]. Symptoms of PBFD, such as beak deformation and feather dystrophy, may not become apparent until the host's immunity weakens [13]. The virus primarily targets the host's immune cells, leading to inflammation of various organs, including the skin, gut, thymus, and bursa of Fabricius [14].

While psittacine birds are the primary hosts of PBFDV, there is a growing number of infections occurring in other bird species. Various bird species from families such as Caprimulgiformes, Coraciiformes, and Passeriformes have been found to be susceptible to PBFDV infection [15]. In 2017, wild parrots, owls, ducks, magpies, and other birds in Australia were identified as carriers of the virus [16]. However, the role of these species as reservoirs of the disease remains understudied.

Budgerigar fledgling disease virus (BFDV; species *Gammapolyomavirus avis* is a highly infectious agent that infects various avian species [17]. It is a member of the genus *Gammapolyomavirus* of the family *Polyomaviridae*, which includes nine other avian polyomaviruses (APVs): Adelie penguin polyomavirus (AdPyV), butcherbird polyomavirus (butcherbird PyV), canary polyomavirus (CaPyV), cormorant polyomavirus (CoPyV), crow polyomavirus (CPyV), Erythrura gouldiae polyomavirus 1 (EgouPyV), finch polyomavirus (FPyV), goose hemorrhagic polyomavirus (GHPyV), and Hungarian finch polyomavirus (HunFPyV) [18]. Although most APVs have a specific host range, BFDV can infect a wide range of avian species, including pet birds such as parrots, domestic birds such as chickens, ducks, and geese, and wild birds such as falcons, gulls, and ostriches [17]. BFDV has a genome of approximately 5 kb, containing six ORFs that encode the large and small tumor antigens, as well as viral proteins (VP) 1-4, which play critical roles in capsid formation and induction of apoptosis [19, 20]. BFDV causes symptoms such as abnormal feather growth, haemorrhage, and lesions in multiple organs, including the heart, liver, and kidneys, which can lead to systemic failure [21]. Subclinical complications occur rapidly, often resulting in sudden death, especially in young birds [21, 22]. Despite being asymptomatic, BFDV hosts can shed the virus for an extended period of up to six months after infection [17–19]. BFDV has been widely reported in captive birds in over 15 countries, including Australia, Germany, Italy, Slovakia, Canada, the United States, Japan, Taiwan, and China [23].

The growing global demand for captive birds has led to an increase in international bird trade, which has become a significant factor in the widespread dissemination of both PBFDV and BFDV, affecting both captive and wild birds [17, 24–26]. For instance, studies have revealed a close link between PBFDV transmission and bird trade patterns through phylogeographic and phylogenetic analysis [25]. These findings emphasize the need for surveillance of both viruses at the local level, especially in countries or regions where bird trading is prevalent. In the past decade, the prevalence and genotypes of PBFDV and BFDV in both captive and wild birds and their phylogenetic relationships to known strains have been studied in numerous countries or regions [3, 27–30]. In particular, risk factors such as young host age, certain host species [31, 32], and sources of sampled birds, i.e., breeding facilities vs. households or veterinary hospitals [33–35], have been identified. However, how these independent factors influence the infectivity and virulence of both viruses has not been clearly established.

Hong Kong is a major importer of captive birds in the international market and is home to a large population of birds sourced from various regions of the world. From 2010 to 2020, over 34 thousand live birds, mostly parrots, were imported to Hong Kong for private, commercial, or breeding purposes under the Convention of International Trade in Endangered Species. In addition, there are an unknown number of illegally trafficked birds that cannot be tracked [36, 37]. These imported birds originate from approximately 30 countries or regions, with a significant number coming from Asia (mainland China, Malaysia, and Singapore), Africa (Mali, Democratic Republic of Congo, and South Africa), South America (Argentina and Guyana), and Europe (Czech Republic, Belgium, and Denmark) [36, 38]. The high density of birds kept in breeding facilities and households in Hong Kong poses a substantial risk of disease transmission and outbreaks [39]. The mixing of pathogenic strains from diverse sources also increases the likelihood of the emergence of new strains that pose a threat to both local captive birds and wild birds that have not yet been exposed to these infectious agents. This is particularly concerning for wild birds in Hong Kong that reside near urban areas, including the critically endangered yellow-crested cockatoo (*Cacatua sulphurea*), which has been introduced into the region [40]. In this study, our objective was to investigate the prevalence and genotypes of PBFDV and BFDV in captive birds in Hong Kong, China. Our goal was to identify the major avian hosts and potential sources of transmission of these viruses.

## Materials and methods

### Collection of fecal samples

A total of 516 fecal samples were collected from captive birds in Hong Kong between November 2019 and January 2022. The samples were obtained from 218 households (n = 346), four pet shops (n = 54), and a veterinary clinic (n = 116). Of the samples collected, 492 were from 43 different parrot species, and the remaining 24 samples were from seven non-parrot species. All samples were collected from individual birds, except for 17 samples from pet shops, which were collected from four cages containing multiple budgerigars (*Melopsittacus undulatus*) or cockatiels (*Nymphicus hollandicus*). All samples were stored in absolute ethanol at -20°C immediately following collection.

### DNA extraction

DNA was extracted from fecal samples using an E.Z.N.A. Stool DNA Kit (Omega Bio-Tek, Norcross, USA) following the manufacturer’s protocol. The extraction process was optimized by using steel beads (5 mm) for homogenization of the sample, followed by treatment with proteinase K (QIAGEN, Hilden, Germany) and disruption using a TissueLyser II (QIAGEN) at 15 Hz for 20 seconds. Each sample was eluted in 40-50 μl of elution buffer. Extracted DNA was stored at -20°C until it was used for experiments. The DNA concentration was measured using a Qubit dsDNA HS Assay Kit (Invitrogen, Waltham, USA).

### Detection of PBFDV and BFDV by PCR

Nested PCR assays were employed to detect the presence of PBFDV and BFDV DNA in fecal samples. Due to sequence mismatches between most of the published primers and reference sequences, we designed new primers for this study, with the exception of OP15, which was described previously by Bert et al. [31, 41]. For primer design, we retrieved over 420 PBFDV and 200 BFDV sequences from the GenBank (NCBI) database and aligned them using Geneious Prime 8.1.9 (www.geneious.com).

To detect PBFDV DNA, two pairs of primers were used in the first PCR to amplify the same region within the replicase-associated protein (*Rep*) gene (reactions A and B; Supplementary Table S1). Each first-round PCR product was then used as a template for nested PCR, in which two independent regions within the template fragment were amplified using two pairs of primers (reactions C and D; Supplementary Table S1). Each reaction mixture for the first PCR included 5 μl of extracted DNA, 0.6 μl of each primer (10 μM; IDT, Coralville, USA), 6 µl 5X of GoTaq Reaction Buffer (Promega, Madison, USA), 0.6 μl of 10 mM dNTP mixture (Invitrogen), 3.6 μl of 25 mM magnesium chloride (Promega), 3 μl of 10% dimethyl sulfoxide (DMSO; Promega), 0.15 μl of bovine serum albumen (BSA; 20 μg/μl; NEB, Ipswich, Suffolk, UK), 0.15 μl of GoTaq polymerase (5 units/μl; Promega), and 15.3 μl of UltraPure DNase/RNase-free distilled water (Invitrogen), making up a total of 30 μl. Each reaction mixture for nested PCR included 1 μl of first-round PCR product, 1.25 μl of each primer (10 μM; IDT), 5 μl of 5X GoTaq Reaction Buffer (Promega), 0.5 μl of 10 mM dNTP mixture (Invitrogen), 3 μl of 25 mM magnesium chloride (Promega), 2.5 μl of 10% DMSO (Promega), 0.125 μl of GoTaq polymerase (5 units/μl; Promega), and 10.375 μl of UltraPure DNase/RNase-free distilled water (Invitrogen), making a total of 25 μl. Touchdown conditions were used for the first PCR, while conventional PCR conditions were used for reactions C and D. The temperature conditions for the first PCR were 95°C for 2 minutes, followed by 43 cycles of 95°C for 30 seconds, 55-48°C (55-49°C for the first 7 cycles and 48°C for the remaining 36 cycles) for 30 seconds, and 72°C for 45 seconds, and a final step of 72°C for 5 minutes. For nested PCR, the conditions were 95°C for 2 minutes, followed by 40 cycles of 95°C for 30 seconds, 48°C for 30 seconds, and 72°C for 45 seconds, and a final step of 72°C for 5 minutes.

To detect BFDV DNA, a pair of primers was designed to amplify the region encoding viral protein (VP) 2/3 and part of VP1 (reaction I; Supplementary Table S2). The resulting PCR product was used as a template for nested PCR (reaction II), using another pair of primers (Supplementary Table S2). To enhance efficiency, 3-μl aliquots from five samples were pooled to serve as templates for the first PCR. Each reaction mixture of the first PCR consisted of 15 μl of pooled extracted DNA, 0.9 μl of each primer (10 μM; IDT), 9 μl of 5X GoTaq Reaction Buffer (Promega), 0.9 μl of 10 mM dNTP mixture (Invitrogen), 5.4 μl of 25 mM magnesium chloride (Promega), 4.5 μl of 10% DMSO (Promega), 0.225 μl of BSA (20 μg/μl; NEB), 0.225 μl of GoTaq polymerase (5 units/μl; Promega), and 13.95 μl of UltraPure DNase/RNase-free distilled water (Invitrogen), making a total of 45 μl. For the nested PCRs, each reaction mixture included 1 μl of the first-round PCR product, 1.25 μl of each primer (10 μM; IDT), 5 μl of 5X GoTaq Reaction Buffer (Promega), 0.5 μl of 10 mM dNTP mixture (Invitrogen), 3 μl of 25 mM magnesium chloride (Promega), 2.5 μl of 10% DMSO (Promega), 0.125 μl of GoTaq polymerase (5 units/μl; Promega), and 10.375 μl of UltraPure DNase/RNase-free distilled water (Invitrogen), making a total of 25 μl. Touchdown PCR was used for the first round, whereas conventional PCR conditions were used for the nested reaction. The temperature conditions for the first PCR were 95°C for 2 minutes, followed by 43 cycles of 95°C for 30 seconds, 58-48°C (58-49°C for the first 10 cycles and 48°C for the remaining 33 cycles) for 30 seconds, and 72°C for 45 seconds, and a final step of 72°C for 5 minutes. For the nested PCR, the conditions were 95°C for 2 minutes, followed by 40 cycles of 95°C for 30 seconds, 48°C for 30 seconds, and 72°C for 30 seconds, and a final step of 72°C for 5 minutes.

For both PBFDV and BFDV detection, the amplified PCR product of the respective viral DNA was used as a positive control, and UltraPure DNase/RNase-free distilled water (Invitrogen) was used as a negative control. Electrophoresis in a 1% agarose gel was used to visualize all PCR products, and subsequent sequencing was performed by BGI (Hong Kong). The identity of the amplified products was verified using BLAST.

### Amplification of the PBFDV and BFDV genomes by rolling-circle amplification

To amplify the complete genome sequences of PBFDV and BFDV, rolling-circle amplification (RCA) was performed using sequence-specific primers (Supplementary Tables S3 and S4) and random hexamer primers (Exo-Resistant Random Primer; Thermo Scientific, Waltham, USA). Highly conserved sies within the two genomes were identified for sequence-specific primer design by aligning reference sequences from the GenBank database, using Geneious Prime 8.1.9 (https://www.geneious.com).

All RCA reactions consisted of two phases. In the first phase, the sequence-specific primers bound to the sample DNA, and in the second phase, the genomes were amplified using phi29 polymerase. For the first phase of RCA, 1 μl of extracted DNA, 1.4 μl of sequence-specific primer mixture (0.1 μl per primer, 10 μM; IDT), 0.5 μl of 10X Phi29 buffer (NEB), and 2.1 μl of UltraPure DNase/RNase-free distilled water (Invitrogen) were mixed. The reaction mixture was then incubated at 95°C for 3 minutes, 50°C for 1 minute, 30°C for 1 minute, and 4°C for 1 minute. After incubation, the mixture was placed on ice and mixed with the following: 2 μl of 10X Phi29 buffer (NEB), 0.2 μl of BSA (20 mg/ml; NEB), 0.133 of 100 mM dNTP (Invitrogen), 0.5 μl of Phi29 DNA polymerase (10,000 U/ml; NEB), 1.888 µl of 500 μM Exo-Resistant Random Primer (Thermo Scientific), and 15.279 μl of UltraPure DNase/RNase-free distilled water (Invitrogen). The mixture was incubated at 30°C for 18 hours and then at 65°C for 10 minutes. All RCA products were electrophoresed in a 1% agarose gel and visualized under UV light. Products that produced bands of the expected sizes were sequenced by BGI.

### Amplification of target genes by PCR

PCR was used to amplify the target genes from PBFDV- and BFDV-positive samples that were not successfully amplified by RCA. The PBFDV *Rep* and *Cap* gene sequences were specifically amplified from the positive samples using four primer pairs. These primers were designed to correspond to conserved regions identified by aligning reference sequences from the GenBank database using Geneious Prime 8.1.9 (Supplementary Table S5). The reaction mixtures consisted of 1 μl of RCA product, 0.9 μl of each primer (10 μM; IDT), 6 μl of 5X GoTaq Reaction Buffer (Promega), 0.6 μl of 10 mM dNTP mixture (Invitrogen), 3 μl of 25 mM magnesium chloride (Promega), 3 μl of 10% DMSO (Promega), 0.15 µl of BSA (20 μg/μl; NEB), 0.15 μl of GoTaq polymerase (5 units/μl; Promega), and 14.3 μl of UltraPure DNase/RNase-free distilled water (Invitrogen), which added up to 30 μl. For all four reactions, touchdown conditions were employed. The temperature conditions were as follows: 95°C for 2 minutes, followed by 40 cycles of 95°C for 30 seconds, 55-51°C (55-52°C for the first 4 cycles and 51°C for the remaining 36 cycles) for 30 seconds, and 72°C for 1.5 minutes, with a final step of 72°C for 5 minutes.

The VP1 and VP2-3 sequences from BFDV-positive samples were amplified using two primer pairs that were designed to correspond to conserved regions identified by aligning reference sequences from the GenBank database using Geneious Prime 8.1.9 (Supplementary Table S6). The reaction mixtures consisted of 1 μl of RCA product, 0.9 μl of each primer (10 μM; IDT), 6 μl of 5X GoTaq Reaction Buffer (Promega), 0.6 μl of 10 mM; dNTP mixture (Invitrogen), 3 μl of 25 mM magnesium chloride (Promega), 3 μl of 10% DMSO (Promega), 0.15 μl of BSA (20 μg/μl; NEB), 0.15 μl of GoTaq polymerase (5 units/μl; Promega), and 14.3 μl of UltraPure DNase/RNase-free distilled water (Invitrogen), totaling 30 μl. Touchdown conditions were used for both reactions. For amplification of VP1, the temperature conditions were 95°C for 2 minutes, followed by 40 cycles of 95°C for 30 seconds, 55-50°C (55-51°C for the first 5 cycles and 50°C for the remaining 35 cycles) for 30 seconds, and 72°C for 1.5 minutes, with a final step of 72°C for 5 minutes. For the amplification of VP2/3, the temperature conditions were 95°C for 2 minutes, followed by 40 cycles of 95°C for 30 seconds, 58.5-52.5°C (58.5-53.5°C for the first 6 cycles and 52.5°C for the remaining 34 cycles) for 30 seconds, and 72°C for 1.5 minutes, with a final step of 72°C for 5 minutes.

The amplified PCR products of the respective viral DNA were used as positive controls, and UltraPure DNase/RNase-free distilled water (Invitrogen) was used as a negative control. All PCR products were visualized by electrophoresis in a 1% agarose gel and subsequently sequenced by BGI. BLAST analysis was used to verify the identity of the amplified products.

### Genetic distance calculation and phylogenetic analysis

Consensus sequences of amplified PBFDV and BFDV DNA segments were generated using Geneious 8.1.9. To create alignment files of the sample and reference sequences, we used MAFFT v7.017 in Geneious 8.1.9 and MEGA X [42]. Genetic distances (Supplementary Tables S7 and S8) were calculated and visualized using Geneious 8.1.9.

Model selection and maximum-likelihood (ML) tree reconstruction (1000 replicates) for PBFDV sequences were performed using IQ-Tree [43–47]. For the *Rep* gene, phylogenies were reconstructed using 871-bp sequences, while for the *Cap* gene, 738-bp sequences were used. The TPM3+I+G4+F model (“3-parameter model”, with a consideration of empirical base frequencies and gamma rate heterogeneity with an allowance of invariable sites) was applied in reconstructing the *Rep* gene phylogeny [48]. For the *Cap* gene, the TN+F+G4 model (Tamura-Nei model with consideration of empirical base frequencies and gamma rate heterogeneity) was used [40]. Reference sequences from previous studies were included [27–49]. The resulting phylogenies were visualized using interactive Tree of Life (iTOL) v6 [50]. Raven circovirus (GenBank ID: DQ146997.1) and gull circoviruses (ID: JQ685854.1 and NC_008521.1) were used as outgroups.

### Survey and risk factor analysis

In addition to the information gathered during sampling, such as age, species and symptoms, information regarding pet owners’ husbandry practices and the living conditions of their birds was also collected (Supplementary Table S9). This information encompassed the medical history of their birds, the types of cages in which they were housed, ventilation conditions, the frequency of cleaning and the cleaning agents used, as well as the frequency of contact with other birds. Complete blood count results for positive birds were provided by the veterinary hospital.

Statistical analysis, including Fisher’s exact test and multiple correspondence analysis (MCA), was performed using R Studio v. 4.0.2 and the FactoMineR and Factoshiny packages for MCA [45, 51–53]. The Hellinger method was used to transform quantitative variables using the decostand function in the vegan package [54, 55].

## Results

### Prevalence of PBFDV

**Fig. 1:**
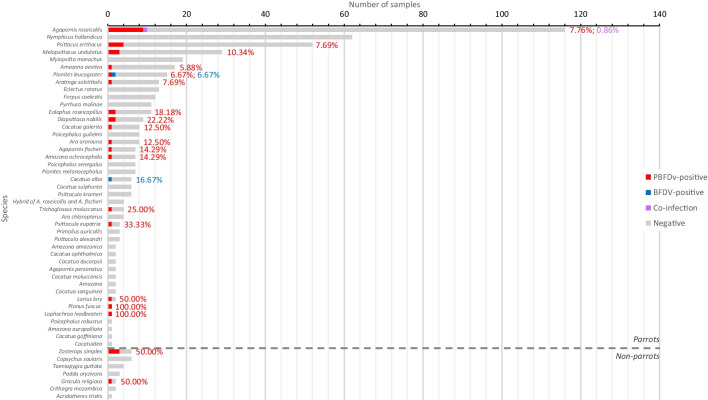
Number of samples tested for PBFDV and BFDV, categorized as positive or negative for each virus, as well as instances of coinfection within each bird species. The percentages of PBFDV infection, BFDV infection, and coinfection are indicated by red, blue, and purple colors, respectively

Out of the 516 fecal samples, PBFDV *Rep* sequences were amplified from 37 (7.17%), using nested PCR (Supplementary Table S10). Of the 37 positive samples, 33 (89.19%) were collected from parrots belonging to 17 different species, while the remaining four (10.26%) were obtained from two non-parrot species (Fig. 1). The prevalence of PBFDV in parrots and non-parrots was found to be 6.71% and 16.67%, respectively. The species with the highest PBFDV prevalence were Major Mitchell’s cockatoo (*Lophochroa leadbeateri*) and dusty parrot (*Aratinga weddellii*), each with a prevalence of 100%. However, it is important to note that only one sample was collected from each of these species. The prevalence values for PBFDV, along with their corresponding 95% confidence intervals, are presented in Supplementary Table S10 [55]. The age of the PBFDV-positive birds sampled in households or animal the clinic ranged from 2.5 months to 15 years.

The prevalence of PBFDV in pet shops (24.07%; 13/54) was significantly higher (Fisher’s exact test, *P* < 0.001) than in households (4.34%; 15/346) and the animal clinic (7.76%; 9/116; Supplementary Table S8). PBFDV was detected in samples of six parrot species obtained from pet shops, including peach-faced lovebirds (*Agapornis roseicollis*), gray parrots (*Psittacus erithacus*), budgerigars, turquoise-fronted amazons (*Amazona aestiva*), yellow-crowned amazons (*Amazona ochrocephala*), and black-capped lories (*Lorius lory*). Additionally, two non-parrot species, Swinhoe’s white-eyes (*Zosterops simplex*) and common hill mynas (*Gracula religiosa*), also tested positive for PBFDV. The rates of PBFDV infection in pet shops ranged from 11.11% to 50.00%.

Of the 36 PBFDV-positive birds for which information regarding symptoms was available, 23 (63.89%) did not exhibit any observable symptoms (Supplementary Table S9). Of the 13 birds (36.11%) that displayed symptoms, five (38.46%) exhibited feather-related symptoms such as feather destructive behavior and feather loss, and one (7.69%) had beak deformation. Other typical signs of sickness in birds, including weight loss and lethargy, were also reported in a few birds, and one bird (7.69%) had digestive symptoms, specifically, diarrhoea (Supplementary Table S11). Using Fisher’s exact test, significant differences in the number of birds with symptoms was observed between the different species (*P* = 0.008), and a weak difference was found in the presence of feather-related symptoms among species (*P* = 0.057; Supplementary Table S11). Notably, none of the 10 positive peach-faced lovebirds exhibited any symptoms.

It was observed that two of the five birds sampled at the animal hospital exhibited hemolysis in their blood in addition to showing feather-destructive behavior. Two other birds developed leukopenia, and one of them died shortly after being admitted to the hospital. The remaining bird showed heteropenia and displayed feather-destructive behavior.

Out of 37 PBFDV-positive birds, only two were found to be suffering from illnesses unrelated to PBFDV, as diagnosed by veterinary practitioners from the animal hospital or based on information provided by the pet owners. One of these, a peach-faced lovebird, had liver disease, and the other, a gray parrot, had uropygial gland impaction (UGI) and died shortly after the stool sample was collected. It was also reported that a PBFDV-positive peach-faced lovebird died within three months after collection of the fecal sample despite not displaying any symptoms.

One of the PBFDV-positive peach-faced lovebirds also tested positive for BFDV, indicating a coinfection. The rate of coinfection with PBFDV and BFDV in our study was 0.194%. At the time of sampling, this bird was experiencing feather loss and displaying signs of feather-destructive behavior. After receiving the test results, the owner responded by adding mineral and vitamin supplements to the bird's regular diet, and the symptoms were alleviated. Half a year later, a second sample was collected, and PBFDV DNA, but not BFDV DNA, was detected in the second fecal sample.

### Prevalence of BFDV

Out of 516 tested, three samples (0.58%) were found to be positive for BFDV DNA (Fig. 1 and Supplementary Table S12). These three samples were collected from different households and belonged to a green-thighed parrot (*Pionites leucogaster*), an umbrella cockatoo (*Cacatua alba*), and a peach-faced lovebird, which was also positive for PBFDV. The age of the BFDV-positive birds ranged from 8 months to 4 years. The peach-faced lovebird, which was coinfected with PBFDV exhibited clinical signs, but the other BFDV-positive birds did not.

### Genetic distances and phylogenetic relationships among PBFDV sequences

Despite our efforts to amplify complete gene sequences from each sample, we were only able to obtain partial sequences for some of them. Specifically, *Rep* sequences ranging from 209 to 871 bp were obtained from 28 PBFDV-positive samples, while *Cap* sequences ranging from 495 to 762 bp were obtained from nine positive samples (Supplementary Tables S13-S15). All sequences have been submitted to and are available in the GenBank database (*Cap*: OR778782-OR778790; *Rep*: OR880917-OR880952).

A unique sequence was successfully amplified from each sample. Out of the 28 positive samples from which the *Rep* gene was amplified, one sample was obtained from pooled fecal samples of two PBFDV-positive peach-faced lovebirds that were housed together in the same cage (ID: 1RAA_2RAA_A.ros_Household). Based on analysis of the chromatograms, no SNPs were found in the amplified sequence(s) from that particular sample, indicating a single *Rep* sequence. The pairwise similarities of the amplified *Rep* and *Cap* gene sequences ranged from 35.9 to 99.9% and from 69.8 to 99.9%, respectively (Supplementary Tables S13-S14).

**Fig. 2:**
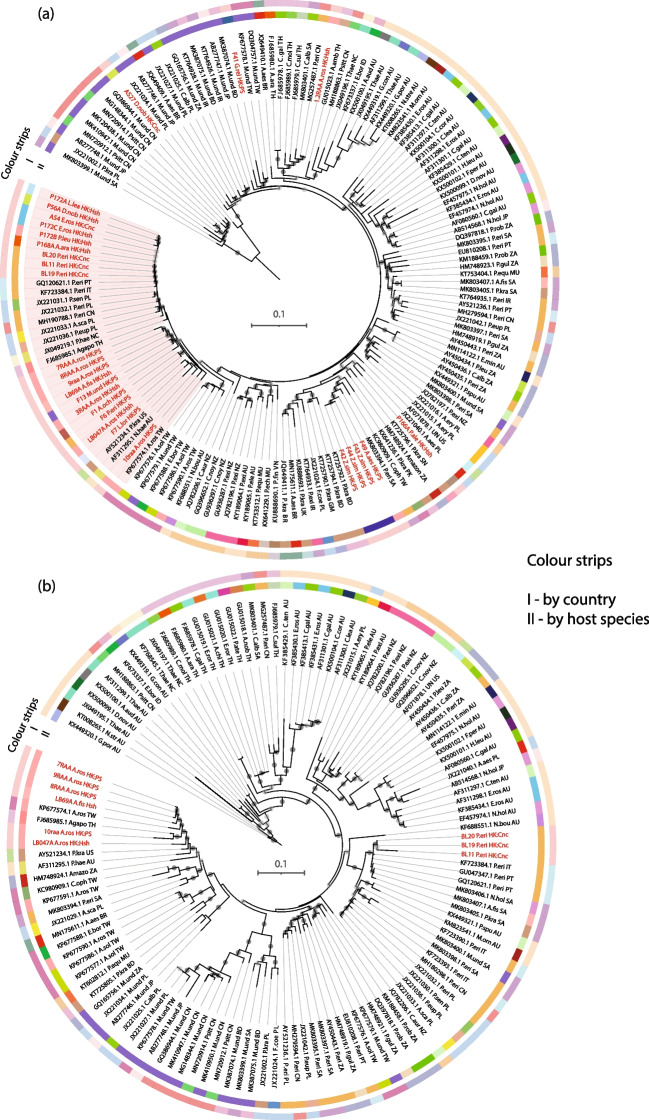
Maximum-likelihood (ML) trees reconstructed using *Rep* and *Cap* gene sequences. (**a**) The *Rep* gene tree includes 871-bp sequences obtained from 28 isolates identified in this study as well as 124 sequences obtained from the GenBank database. The monophyletic group that includes most of the Hong Kong sequences is highlighted in pink. (**b**) The *Cap* gene tree includes 738-bp sequences obtained from nine individuals in Hong Kong as well as 119 sequences from GenBank. A list of the sequences included in the tree is provided in Supplementary Table S10. The trees also included raven circovirus (DQ146997.1) and gull circovirus (JQ685854.1 and NC_008521.1) sequences as outgroups (Supplementary Fig. S1). The sequences from this study are indicated by red color labels. In both trees, the outer color strips represent the country of origin of the isolates, while the inner color strips represent the host species. The tip labels also include the country of origin and host species, denoted as the country code and short forms (e.g., P. eri: *Psittacus erithacus*), respectively. Bootstrap values between 70 and 90 are indicated by light gray circles, while bootstrap values >90 are indicated by dark gray circles. Geographical distribution of PBFDV sequences closely related to those in Hong Kong can be found in Supplementary Fig. S2

ML trees were reconstructed using the amplified sequences obtained from our samples, along with other PBFDV sequences obtained from the GenBank database (Fig. 2). In the *Rep* gene phylogeny, most of the sequences obtained from our samples formed a monophyletic group (Fig. 2). Within this clade, distinct groupings based on the host species were observed, particularly among lovebirds (*Agapornis*) and gray parrots. One subgroup primarily consisted of sequences collected from peach-faced lovebirds and Fischer’s lovebirds (Fig. 2), along with some isolates obtained from other parrots in Hong Kong. Additionally, a few sequences from Taiwan, the USA, and Australia also clustered within this subgroup. The other subgroup mainly comprised isolates from gray parrots collected in Hong Kong, mainland China, Poland, and Italy. Moreover, several sequences collected from various parrot species in Hong Kong formed a separate cluster within this subclade. These isolates showed close genetic relatedness to isolates from Poland, New Caledonia, and Thailand.

Apart from those clustered within the previously mentioned monophyletic group, *Rep* gene sequences obtained from Swinhoe’s white-eyes (GenBank accession number: OR880946) and yellow-crowned amazon (GenBank accession number: OR880940), sampled from the same shop, were closely grouped together in another highly supported clade with sequences from Saudi Arabia, Taiwan, and Pakistan. Other isolates from Hong Kong were dispersed throughout other parts of the *Rep* gene tree, displaying close genetic relatedness to isolates from various host species and locations, including mainland China, Saudi Arabia, Taiwan, Bangladesh, Japan, Thailand, Pakistan, and even Poland.

The phylogenetic tree based on the *Cap* sequences exhibited a different topology. Rather than clustering together, our sequences were divided into two distantly related clusters, with each consisting of PBFDV sequences from the same host species, either gray parrot or rosy-faced lovebird. These sequences originated from different countries or regions. Specifically, sequences obtained from lovebirds in Hong Kong formed a cluster with sequences from Thailand and Taiwan with a high bootstrap value. In another distantly related branch, three isolates from gray parrots clustered together with sequences from Italy, Poland, and Portugal.

### BFDV sequences

Partial sequences of the BFDV VP2/3 gene were obtained by PCR, with sequences ranging in length from 335 to 336 bp. The nucleotide sequences of the isolates were similar to each other (99.2 to 100% identity) and to other sequences in the GenBank database (97.5 to 100% identity; Supplementary Table S15). All sequences have been submitted to and are available in the GenBank database (OR778883-OR778885).

### Multiple correspondence analysis of potential risk factors associated with the presence of PBFDV

The husbandry information for 221 birds from households, along with the information recorded by samplers, was analyzed using MCA to investigate potential associations between categorical variables and the presence of PBFDV. Since the variables of husbandry practice, medical background, and frequencies of social activities were recorded for the birds from households only, the analyses were done with two datasets, one of which only included the birds from households and the other of which included data from all of the birds.

The MCA results of the dataset including birds from households did not reveal any clear patterns or associations between variables (Supplementary Figs. S3, S4). Dimension 1 was primarily influenced by the agents used for cage cleaning, while dimension 2 was primarily influenced by the species of the birds. In terms of the presence or absence of PBFDV in our samples, the points were close to the origin, indicating minimal variation in this variable.

The MCA results for the dataset including birds from all sources revealed that the presence of PBFDV was weakly associated with dimension 1 (Supplementary Figs. S5, S6), which was primarily influenced by the source of the bird (i.e., pet shop, household, or animal clinic). This observation was in line with the results obtained using Fisher’s exact test discussed above. Although an association was suggested by MCA (Supplementary Figs. S3-S6), the difference in the infection rate between parrots and non-parrots was not statistically significant according to Fisher’s exact test (*P* = 0.091). The MCA results did not reveal any association between other input variables, such as the season and month of sample collection (Supplementary Tables S16-21).

## Discussion

A total of 516 bird samples from households, pet shops, and an animal clinic were analyzed to determine the prevalence and genotypes of PBFDV and BFDV in captive birds in Hong Kong. PBFDV and BFDV were detected in 7.36% and 0.57% of these samples, respectively. These rates are lower than those reported in other parts of Asia [23, 26, 28, 33–35, 56]. PBFDV was detected in 24.07% of the samples from pet shops in Hong Kong, which is similar to the reported prevalence in breeding facilities in Eastern Turkey (23.00%) [28] but lower than that in Bangladesh (54.05%) [33], Fuzhou, China (80.00%) [34], and Taiwan (29.7% and 41.18%) [57, 59]. No BFDV-positive samples were found in pet shops, which is in contrast to reports from Sichuan China [29], Japan [59], and Eastern Turkey [28], where infection rates ranged from 2.7% to 100%. The prevalence of PBFDV and BFDV in households in Hong Kong was also lower than in Taiwan, which was 21.7%-41.2% and 8.3%-15.2%, respectively, according to different studies [35, 57, 58]. The infection rates of these two viruses in veterinary facilities have been reported to be 37.84% and 4.61% for PBFDV and BFDV in Bangladesh and South Korea, respectively [23, 33], which are higher than the rates in Hong Kong.

Our findings indicate significant differences in PBFDV infection rates among the different sample sources. Particularly noteworthy is the high infection rate observed in pet shops, which is not surprising, considering that traded birds are often key vectors for transmission of PBFDV across borders [11, 24, 25]. Furthermore, pet shops or breeding houses serve as hubs that gather a large number of imported or trafficked birds from diverse origins, creating environments conducive to the mixing and spread of pathogens [60, 61]. The transmission of pathogens through physical contact among birds is particularly likely in commercial settings, due to the breeding practices that are employed in aviaries [58]. Various other factors related to the living conditions in pet shops, such as poor hygiene, high bird density, rapid turnover of birds, and frequent disturbances in the environment, are also contributing risk factors for diseases in birds in general [39, 60]. We observed significant differences in PBFDV infection rates and symptoms among different genera and species of birds. Previous studies have suggested that the susceptibility to PBFDV infection and the severity of symptoms may vary depending on host species. For instance, gray parrots are thought to be more susceptible to PBFDV infection and more likely to exhibit severe symptoms than other species [30, 62, 63]. However, in our study, we did not find a particularly high infection rate or more-severe symptoms in gray parrots. PBFDV DNA was detected in 7.69% of gray parrots from 33 sampling sites, which was actually lower than the average infection rate for all bird species (i.e., 10.98%). Moreover, half of the positive gray parrots did not show any observable clinical signs. On the other hand, we observed a significantly low PBFDV prevalence in cockatiels. Despite sampling over 60 cockatiels from 31 different living units, none of them tested positive. This finding aligns with previous studies [64–66] in which a surprisingly low incidence rate of PBFDV infection was consistently found in cockatiels, despite their popularity as pets. However, these findings contrast with the results of PBFDV surveillance in Iran, which suggested that over 35% of the captive cockatiel population in that country may have been infected [32]. These contradictory observations indicate that the relationship between host species and PBFDV pathology may be more complex than previously understood. Varsani et al. reported that the different PBFDV genotypes exhibit a wide range of both host specificity and geographical distribution worldwide [15]. Therefore, it is likely that there is significant variability in susceptibility to PBFDV, and the symptoms exhibited by different bird species when exposed to different PBFDV genotypes in different geographical locations.

Overall, PBFDV was detected in 17 out of 43 (39.53%) sampled parrot species, suggesting a significant infection rate among parrot species in Hong Kong. Interestingly, PBFDV was also found in two non-parrot species, namely the Swinhoe’s white-eye and the common hill myna, with a high infection rate of 50% each. This represents the second known instance of PBFDV in common hill mynas, with the first report dating back two decades in a captive flock in Germany [67]. Additionally, our study is the first to document PBFDV in Swinhoe’s white-eyes or any member of the genus *Zosterops*, indicating a broader host range for PBFDV than previously described. Notably, since we also detected PBFDV in a turquoise-fronted amazon in the same shop, it is likely that the virus strains in found non-parrot birds were transmitted from parrots that were kept in close proximity.

The discovery of PBFDV strains capable of infecting both psittacines and passerines in Hong Kong raises serious ecological concerns. In this densely populated city, the proximity of urban wildlife and humans increases the likelihood of virus spillover from captive to wild bird populations [68, 69]. This is particularly problematic in open-air pet shops, where wild birds can come into close contact with shop animals and amenities when attracted by spilt food and water. Captive birds in pet shops occasionally escape from their cages and even establish feral populations in the surroundings [70]. There is a risk that PBFDV could be transmitted to wild birds via infected captive birds, which may serve as a reservoir, or via environmental contamination. In addition to the critically endangered yellow-crested cockatoos that have been introduced into Hong Kong, the city is also home to numerous ecologically significant and threatened non-parrot avian species [71]. Given that the effects of PBFDV on non-psittacine hosts remain largely unknown, the ecological impact of the virus on wildlife in Hong Kong should not be underestimated.

To investigate the phylogenetic relationships between PBFDV strains found in our samples and previously discovered strains from other regions, ML trees were reconstructed using *Rep* and *Cap* gene sequences. Comparing the phylogenies reconstructed using the *Cap* sequences obtained in this study and sequences from other regions, we observed differences in clustering patterns and topologies between the two trees. Notably, the branches in the *Cap* tree exhibited greater elaboration than those in the *Rep* tree, suggesting that the *Cap* gene has undergone more genetic changes and evolved at a faster rate than the *Rep* gene. This finding is consistent with conclusions drawn from previous studies [32, 72], which highlight the higher mutation and recombination rates in the *Cap* gene. These factors could potentially drive host switching in PBFDV [72].

Our sequence analysis revealed distinct clustering patterns in the two phylogenies. In the *Rep* tree, most of our sequences clustered together in a monophyletic clade, suggesting that the evolution of the *Rep* gene in PBFDV in Hong Kong may be more influenced by location or origin. These findings are consistent with those reported by Fogell et al. [49], who similarly observed that the majority of clades in the *Rep* phylogeny were monophyletic by location. On the other hand, our *Cap* tree displayed clustering based on host species, which aligns with the findings in Iran [32]. These distinct clustering patterns can be explained by differences in the functional roles of the expressed proteins and the selective pressures that influenced the evolution of the two genes. The capsid protein, serving as the antigen for host immune recognition, is subjected to strong purifying and positive selection [5, 73]. This intense selection can result in the generation of diverse host-based genotypes, as observed in rainbow lorikeet PBFDV *Cap* genotypes and those of other circoviruses [73]. In contrast, the *Rep* gene encodes the replicase-associated protein, which is necessary for replication of the viral genome. Rather than selective forces associated with host species, the *Rep* gene is believed to be more susceptible to purifying selection due to its vital function in virus replication [5, 73]. Additionally, the evolution of the *Rep* gene is likely driven by frequent recombination [5] and genetic drift [74], both of which occur through the random sampling of viral strains between traded birds during their frequent mixing and distribution across counties or regions involved in trade [75]. Although host species-associated clustering is evident in the *Cap* phylogeny, multiple clades in the tree contain sequences from distantly related host species, suggesting that these strains may have a broader host range and could be host generalists.

The *Rep* gene sequences of PBFDV strains with the same geographical background tend to cluster together, making it more reliable to determine the possible origins of strains in Hong Kong based on the *Rep* gene clustering pattern. According to the *Rep* phylogeny, PBFDV strains in Hong Kong showed close relationships to strains from Europe, including Poland and Italy, as well as other parts of Asia, including mainland China, Thailand, Taiwan, Saudi Arabia, Bangladesh, and Pakistan. It is likely that PBFDV strains in Hong Kong originated in Europe, since several European countries, including the Czech Republic, Belgium, and Denmark, have been major exporters of pet birds to Hong Kong [36]. Although our sequences did not cluster with PBFDV sequences from these specific countries, they did cluster with strains from Poland, which is consistent with global PBFDV transmission patterns [25] and with a phylodynamic and phylogeographic study that demonstrated the dispersion of PBFDV from Europe to parts of Asia during the 2010s through the parrot trade [76]. A previous survey showed that a large number of pet parrots in Hong Kong were illegally trafficked from mainland China [38], which could be one of the routes by which PBFDV was introduced into Hong Kong. The finding that PBFDV strains in other Asian countries or regions are closely related to those in Hong Kong, despite little or no known trading relationship, suggests that they may share similar origins in Europe and mainland China [36].

Finally, our study did not find any significant PBFD risk factors associated with the season of sampling, husbandry practices, health history of the bird, cage type, or presence of ventilation. However, it is important to note that the reliability of these results is limited by the small number of PBFDV-positive samples in our study. It is worth mentioning that the implementation of hygiene measures has been shown to effectively reduce the infection rate of PBFDV in wild echo parakeets [77]. Therefore, it is hoped that future research will provide information on the effect of hygiene practices on PBFDV transmission among captive birds.

In conclusion, we have identified the presence of both PBFDV and BFDV in captive birds in Hong Kong, originating from multiple sources. Although the prevalence of these viruses was lower than in nearby regions, it is crucial not to underestimate their potential impact on local bird populations, particularly PBFDV, which was detected not only in parrots but also in other avian species. Considering the potential ecological consequences of these viruses in the wildlife of Hong Kong and the need to protect the welfare of captive animals, we strongly recommend that local authorities regularly conduct surveillance for PBFDV and BFDV in both wild and captive birds. Additionally, measures should be taken to enhance the separation between captive and wild animals, such as creating enclosed environments in pet shops to prevent contact between wild and captive birds. Furthermore, we suggest implementing molecular testing and clinical examinations targeting PBFDV and BFDV, in addition to the current screening for zoonotic pathogens, for imported birds to prevent the introduction of these viruses [78]. Given the ease with which the boundary between wild and captive animal populations can be breached in urban areas like Hong Kong through accidental releases and environmental contamination [67], it is vital to establish local monitoring and tracking systems for animal pathogens to prevent and mitigate the effects caused by these infectious agents.

### Supplementary Information

Below is the link to the electronic supplementary material.

Supplementary file1 (PDF 3.04 MB)

Supplementary file2 (XLSX 141 KB)

Supplementary file3 (XLSX 79 KB)

## Data Availability

The sequences generated in this study are available in the GenBank database. PBFDV sequences can be found under accession numbers (*Cap*) OR778782-OR778790 and (*Rep*) OR880917-OR880952. BFDV sequences can be found under accession numbers OR778883-OR778885.
