# Supplementary material for: Prevalence, genotypes, and infection risk factors of psittacine beak and feather disease virus and budgerigar fledgling disease virus in captive birds in Hong Kong
Source: Arch Virol. 2024 Apr 5;169(5):91. doi: 10.1007/s00705-024-06017-3 (PMC10997714; doi:10.1007/s00705-024-06017-3)
Supplement: Supplementary file 1 — Supplementary file1 (PDF 3.04 MB) [file 705_2024_6017_MOESM1_ESM.pdf]

Supplementary Material 1 for

Prevalence, genotypes, and infection risk factors of psittacine beak and feather disease virus and budgerigar fledgling disease virus in captive birds of Hong Kong

Table S1-2.	Primers used in detection PCRs
Table S3-4.	Designed sequence specific primers used in Rolling-circle amplification (RCA)
Table S5-6.	Designed primers used in PBFDV & BFDV gene fragment amplification
Table S7-8.	Reference sequences used in primers design, tree construction, and genetic distance calculations
Table S9.	Survey responses
Table S10.	Number of PBFDV-positive fecal samples per species from three respective sources
Table S11.	Symptom statistics of each species
Table S12.	Number of BFDV-positive fecal samples per species from three respective sources
Figure S1.	Phylogenetic trees with outgroups
Figure S2.	World map indicating countries or regions with PBFDV sequences closely related to Hong Kong sequences
Figure S3-6.	MCA results

Table S1. Designed primer sequences targeting the *Rep* gene for PBFDV DNA detection.

First step of nested PCR				
**Reaction**	Primer name	Direction	Primer sequence (5' to 3') #	Template
**A***	F3	Forward	TATGYCATCGTTGGACG	Fecal DNA
	OP15	Reverse	GTCACAGTCCTCCTTGTACC	
**B***	F3	Forward	TATGYCATCGTTGGACG	Fecal DNA
	R2	Reverse	AGGCGGAGCATCTCGCAATA	
Second step of nested PCR				
**Reaction**	Primer name	Direction	Primer sequence (5' to 3') #	Template
**C**	F4	Forward	GCCTGTAGGGAACCCAGC	Reaction A & B PCR products*
	R2	Reverse	AGGCGGAGCATCTCGCAATA	
**D**	F3	Forward	TATGYCATCGTTGGACG	Reaction A & B PCR products*
	R1	Reverse	TCTCCGCCACAATGCC	

# All primers were designed manually for this study except OP15 was designed by Ypelaar et al., 1999 [32].

* Products of both reactions A and B were used as templates for the nested reactions C and D.

Table S2. Designed primer sequences targeting the *vp1* and *vp2/3* genes for BFDV DNA detection.

First step of nested PCR				
**Reaction**	Primer name	Direction	Primer sequence (5' to 3') #	Template
**I**	APV_1509F	Forward	AGCCCTGATTGTGTACCAAAGT	Fecal DNA
	APV_2167R	Reverse	CGGTATCCTGGCACAGCT	
Second step of nested PCR				
**Reaction**	Primer name	Direction	Primer sequence (5' to 3') #	Template
**II**	APV_1568F	Forward	CCGCACGTTGGACATTAACA	Reaction I PCR products
	APV_1927R	Reverse	GGTACCTGCTGAGGTCGTG	

All primers were designed manually for this study.

Table S3. PBFDV genome-specific primers used in RCA. Bases with asterisks (*) in the front were treated with phosphorothioate bond modifications.

Primer names	Sequence
pbfd_rca_r1_F1	CTGGTACAAGGAGGAC*T*G
pbfd_rca_r1_F2	CCAATGAGCAGCCTG*G*G
pbfd_rca_r1_F3	GAG ATG CTC CGC CTC*T*G
pbfd_rca_r1_R1	GACTTCCTTCATTTTGC*G*T
pbfd_rca_r1_R2	GTACCTTGCTCGCCCTT*T*T
pbfd_rca_r1_R3	TCTCGCCACAATG*C*C
pbfd_rca_r1_F4	ACGAGCAAT*A*A
pbfd_rca_r2_F1	CGAAATTCAGTGTATGTGGG*T*T
pbfd_rca_r2_F2	GGGTCTTGGTTTTGGTCT*G*C
pbfd_rca_r2_F3	ATTGGCGTTTGAATCT*G*A
pbfd_rca_r2_R1	GTGGCTAAACAGT*G*C
pbfd_rca_r2_R2	ATGTTCAATTCCGTCAATT*T*G
pbfd_rca_r2_R3	CAAACGCCTCCTCA*G*A
rca_pbfd_r2_f4	ATTATAAAATG*C*G

Table S4. BFDV genome-specific primers used in RCA. Bases with asterisks (*) in the front were treated with phosphorothioate bond modifications.

Primer names	Sequence
apv_rca_r1_F1	CAAATGACTGGTTACTC*C*C
apv_rca_r1_F2	TACCTCGCCTCCTAG*T*A
apv_rca_r1_F3	AGAACAGCAAACCCC*C*C
apv_rca_r1_R1	TACTTTGGTACACAATCA*G*G
apv_rca_r1_R2	TATCAGGGATGGGGCACT*T*A
apv_rca_r1_R3	CGAGAACCATTCCGCT*C*C
apv_rca_r1_F4	TACAAGATGTAT*T*A
apv_rca_r1_R4	GTTCCATATAC*T*T
apv_rca_r1_f5	CGATTTA*T*A
apv_rca_r2_F1	GCAAGTAACAATAGGGC*A*T
apv_rca_r2_F2	CCACAACATTCATATCAATAC*T*G
apv_rca_r2_F3	CTTCACCGTGTCCTTGT*C*C
apv_rca_r2_R1	GCTAGAAAAACCGAC*G*C
apv_rca_r2_R2	CCAATGTGCTGTGGATG*C*T

Table S5. Designed primer sequences for amplification of *Rep* and *Cap* gene segments from PBFDV-positive samples.

Amplified sequences	Reaction	Primer name	Direction	Primer sequence (5' to 3') #
***Rep* + *Cap* partial sequences**	B1	P_386F	Forward	AATTCYACTATGCCATCGTTGG
		P_1774R	Reverse	GGRTTCAAACGMCTCCTCAG
	B2	P_2142F	Forward	CTATTGGTYGWGAADCGGCGTCTGC
		P_622R	Reverse	CGGTGACCRTCTCKCGCCAC
	B3	P_1669F	Forward	GGTCCTCCTTGTAGTGGGATC
		P_583R	Reverse	AYRTCBCCYTCYTTRCTRCA
***Rep* partial sequence**	B4	P_877F	Forward	GGKGARTGGTGGGAYGGVT
		P_1654R	Reverse	CGAAGCTGAAGGCAATGCCG

All primers were designed manually for this study.

Table S6. Designed primer sequences for amplification of *vp1* and *vp2-3* from BFDV-positive samples.

Amplified sequences	Primer name	Direction	Primer sequence (5' to 3') #
VP1	B_1869F	Forward	GGACCTGACTCCAGAGTGG
	B_3939R	Reverse	TATTTAGCGGGGAGCTTTGG
VP2-3	B_810F	Forward	TGTCCCTCCTTCACAGCCTA
	B_2167R	Reverse	CGGTATCCTGGCACAGCT

All primers were designed manually for this study.

Table S7. Reference sequences used in PBFDV primers design, tree construction, and genetic distance calculations

GenBank accession	Year	Country / region	Host species	GenBank accession	Year	Country / region	Host species
AB277746	2010	JP	*Melopsittacus undulatus*	FJ685980	2006	TH	*Ara ararauna*
AB277747	2016	JP	*Melopsittacus undulatus*	FJ685985	2005	TH	*Agapornis* sp.
AB277748	2010	JP	*Melopsittacus undulatus*	FJ685989	2006	TH	*Cacatua moluccensis*
AB514568	2010	JP	*Nymphicus hollandicus*	GQ120621	2008	PT	*Psittacus erithacus*
AF071878	1998	US	Unknown	GQ165756	2003	ZA	*Melopsittacus undulatus*
AF080560	1998	AU	*Cacatua galerita*	GQ386944	2008	CN	*Melopsittacus undulatus*
AF311295	2001	AU	*Psephotus haematogaster*	GQ396652	2008	NZ	*Cyanoramphus novaezelandiae*
AF311297	2001	AU	*Cacatua tenuirostris*	GU015018	2006	TH	*Ara nobilis*
AF311298	2001	AU	*Eolophus roseicapillus*	GU015019	2006	TH	*Eclectus roratus*
AF311299	2001	AU	*Trichoglossus haematodus*	GU015020	2006	TH	*Eclectus roratus*
AF311300	2001	AU	*Cacatua leabeateri*	GU015021	2009	TH	*Ara chloropterus*
AF311301	2001	AU	*Cacatua galerita*	GU015022	2006	TH	*Probosciger aterrimus*
AY450434	2004	ZA	*Pionites leucogaster*	GU015023	2006	TH	*Ara ambigua*
AY450435	2004	ZA	*Psittacus erithacus*	GU047347	2009	PT	*Psittacus erithacus*
AY450436	2004	ZA	*Cacatua alba*	GU936287	2008	NZ	*Platycercus eximius*
AY450443	2004	ZA	*Psittacus erithacus*	GU936295	2008	NZ	*Cyanoramphus novaezelandiae*
AY521234	2016	US	*Psittacula krameri*	GU936297	2008	NZ	*Cyanoramphus novaezelandiae*
AY521236	2016	PT	*Psittacus erithacus*	HM748919	2008	ZA	*Poicephalus gulielmi*
DQ304757	2016	TW	*Melopsittacus undulatus*	HM748921	2008	ZA	*Poicephalus gulielmi*
DQ397818	2006	ZA	*Poicephalus robustus*	HM748923	2008	ZA	*Poicephalus gulielmi*
EF457974	2008	AU	*Nymphicus hollandicus*	HM748924	2008	ZA	*Amazon* sp.
EF457975	2008	AU	*Nymphicus hollandicus*	JQ649409	2011	BR	*Amazona aestiva*
EU810208	2005	PT	*Psittacus erithacus*	JQ782196	2009	NZ	*Platycercus eximius*
FJ685978	2005	TH	*Cacatua galerita*	JQ782206_	2012	NZ	*Cyanoramphus auriceps*
FJ685979	2011	TH	*Cacatua sulphurea*	JX049197	2011	NC	*Trichoglossus haematodus*

GenBank accession	Year	Country / region	Host species	GenBank accession	Year	Country / region	Host species
JQ649410	2011	BR	*Amazona aestiva*	KF385434	2004	AU	*Eolophus roseicapillus*
JQ649411	2011	BR	*Psittacula krameri*	KF673337	2005	ID	*Eos bornea*
JQ782196	2009	NZ	*Platycercus eximius*	KF688551	1996	AU	*Neopsephotus bourkii*
JQ782197	2009	NZ	*Platycercus eximius*	KF723384	2009	IT	*Psittacus erithacus*
JQ782200	2009	NZ	*Platycercus eximius*	KF723390	2011	IT	*Psittacus erithacus*
JQ782206	2012	NZ	*Cyanoramphus auriceps*	KF723393	2009	IT	*Psittacus erithacus*
JX049195	2009	AU	*Trichoglossus haematodus*	KF768545	2013	NC	*Trichoglossus haematodus*
JX049196	2011	AU	*Trichoglossus haematodus*	KM188458	2011	ZA	*Poicephalus robustus*
JX049219	2011	NC	*Psephotus haematogaster*	KM188459	2011	ZA	*Poicephalus robustus*
JX221002	2010	PL	*Psittacula krameri*	KM823541	2014	AU	*Merops ornatus*
JX221015	2006	PL	*Aprosmictus erythropterus*	KP677574	2016	TW	*Agapornis roseicollis*
JX221016	2006	PL	*Aprosmictus erythropterus*	KP677576	2016	TW	*Aratinga solstitialis*
JX221024	2007	PL	*Forpus coelestis*	KP677577	2016	TW	*Aratinga solstitialis*
JX221025	2007	PL	*Cacatua alba*	KP677578	2016	TW	*Melopsittacus undulatus*
JX221027	2007	PL	*Melopsittacus undulatus*	KP677579	2016	TW	*Melopsittacus undulatus*
JX221029	2008	PL	*Alisterus scapularis*	KP677586	2016	TW	*Aratinga solstitialis*
JX221030	2008	PL	*Poicephalus senegalus*	KP677588	2016	TW	*Eos bornea*
JX221031	2008	PL	*Poicephalus senegalus*	KP677590	2016	TW	*Agapornis roseicollis*
JX221032	2008	PL	*Psittacus erithacus*	KP677591	2016	TW	*Agapornis roseicollis*
JX221033	2008	PL	*Alisterus scapularis*	KT008265	2014	AU	*Ninox strenua*
JX221034	2008	PL	*Melopsittacus undulatus*	KT602812	2014	MU	*Psittacula eques*
JX221036	2008	PL	*Psittacula eupatria*	KT725790	2014	GM	*Psittacula krameri*
JX221040	2008	PL	*Amazona aestiva*	KT725792	2014	BD	*Psittacula krameri*
JX221042	2009	PL	*Psittacula eupatria*	KT725794	2014	BD	*Psittacula krameri*
KC980909	2013	TW	*Cacatua ophthalmica*	KT725796	2015	SN	*Psittacula krameri*
KF385413	2005	AU	*Cacatua galerita*	KT725805_	2014	BD	*Psittacula krameri*
KF385429	2013	AU	*Cacatua tenuirostris*	KT753404	2013	MU	*Psittacula eques*
KF385430	2012	AU	*Eolophus roseicapillus*	KT753512	2015	MU	*Psittacula eques*
KF385431	2004	AU	*Eolophus roseicapillus*	KT764926	2014	IR	*Melopsittacus undulatus*

GenBank accession	Year	Country / region	Host species
KT764928	2014	IR	*Melopsittacus undulatus*
KT764933	2014	IR	*Platycercus eximius*
KT764935	2014	IR	*Platycercus eximius*
KU888690	2015	VN	*Psittacula finschii*
KU888693	2013	UK	*Psittacula krameri*
KX449319	2015	AU	*Glossopsitta concinna*
KX449320	2015	AU	*Glossopsitta porphyrocephala*
KX449321	2015	AU	*Purpureicephalus spurius*
KX500099	2014	AU	*Dacelo novaeguineae*
KX500100	2015	AU	*Aquila audax*
KX500101	2015	AU	*Haliaeetus leucogaster*
KX500102	2015	AU	*Falco peregrinus*
KX500104	2015	AU	*Corvus coronoides*
KX641229	2011	MU	*Psittacula echo*
KX641236	2014	PK	*Psittacula krameri*
KY189064	2015	AU	*Platycercus eximius*
KY189065	2015	AU	*Platycercus elegans*
MG148344	2016	CN	*Melopsittacus undulatus*
MG257487	2016	CN	*Psittacus erithacus*
MH180298	2017	CN	*Psittacus erithacus*
MH188863	2017	CN	*Psittaciformes*
MH190788	2017	CN	*Psittacus erithacus*
MH279594	2017	CN	*Psittacus erithacus*
MK120438	2018	CN	*Melopsittacus undulatus*
MK387074	2017	BD	*Melopsittacus undulatus*
MK387075	2017	BD	*Melopsittacus undulatus*
MK410947	2018	CN	*Melopsittacus undulatus*

GenBank accession	Year	Country / region	Host species
MK410950	2018	CN	*Melopsittacus undulatus*
MK803394.1_	2018	SA	*Psittacus erithacus*
MK803395.1	2017	SA	*Psittacus erithacus*
MK803397.1	2017	SA	*Psittacus erithacus*
MK803398	2017	SA	*Psittacus erithacus*
MK803399	2017	SA	*Melopsittacus undulatus*
MK803400	2017	SA	*Melopsittacus undulatus*
MK803401	2017	SA	*Cacatua alba*
MK803405	2018	SA	*Psittacula krameri*
MK803406	2018	SA	*Nymphicus hollandicus*
MK803407	2018	SA	*Agapornis fischeri*
MN114122	2017	AU	*Eudyptula minor*
MN175611	2016	BR	*Amazona aestiva*
MN720912	2018	CN	Psittaciformes
MN720914	2018	CN	Psittaciformes

Table S8. Reference sequences used in BFDV primers design and genetic distance calculations

GenBank accession	Year	Country / region	Host species
AB453159	2009	Unknown	Unknown
AB453161	2009	Unknown	Unknown
AB453162	2009	Unknown	Unknown
AB453163	2009	Unknown	Unknown
AB453165	2009	Unknown	Unknown
AB477106	2009	JP	Unknown
AF118150	2003	Unknown	*Pteroglossus viridis*
AF241168	2010	Unknown	Unknown
AY672646	2008	CN	*Melopsittacus undulatus*
FJ385773	2008	CN	*Psittacula* sp.
GU452537	2012	CN	*Melopsittacus undulatus*
KT203762	2009	PL	*Psittacula eupatria*
KT203764	2010	PL	*Psittacula krameri*
KT203765	2010	PL	*Melopsittacus undulatus*
KT203768	2010	PL	*Melopsittacus undulatus*
KX008968	2015	PT	*Ara chloroptera*
M20775	2003	Unknown	Unknown
MH643735	2018	CN	*Melopsittacus undulatus*
MK516256	2014	South Korea	*Psittacula eupatria*
NC_004764	2018	Unknown	Unknown

Table S9. Survey responses

Section	Question	No. of responses	Response
Background	Source of bird	218	Pet shop: 50.45%<br>Self-bred: 22.02%<br>Other owners: 12.84%<br>Abandoned pets / Animal shelter: 8.25%<br>Breeder: 5.05%<br>Others: 0.92%<br>Unknown: 0.46%
	Origin of bird	110	Hong Kong: 83.64%<br>Australia and the Philippines: 3.64%<br>Mainland China: 2.73%<br>South Africa: 1.82%<br>Europe, Indonesia, Macao, Netherlands & Taiwan: 0.91%
	Medical history	221	No: 80.09%<br>Yes: 19.91% (e.g., feather-biting, digestive symptoms, infertility, *Chlamydia psittaci* infection, flea, bird flu, imbalance, pbfd, sinus)
	Vaccination	221	No: 99.55%<br>Yes (PBFD): 0.45%
Husbandry	Frequency of changing feed	216	Ranging from every 7 hours to once per half-year<br>Average: every 2 days
	Frequency of changing water	217	Ranging from every 4 hours to once per week<br>Average: once per a day and a half
	Any vitamin or mineral supplements	221	Vitamins only: 6.33%<br>Minerals only: 18.55%<br>Both vitamins and minerals: 31.22%<br>None: 43.89%
	Frequency of cleaning	201	Ranging from every 5 and a half hours to once per week<br>Average: once per a day and a half
	Reagent(s) used for cleaning (not mutually exclusive)	221	Water: 46.61%<br>Hot water: 6.79%<br>Detergent: 29.00%

			Alcohol: 9.95%<br>Pet-friendly chemicals (PFC): 2.26%<br>Bleach: 1.81%<br>Unknown: 5.88%
Living environment	With ventilation or not	221	Yes: 96.38%<br>No: 3.62%
	Caged or not	219	Yes: 94.53%<br>No: 5.47%
	Cage volume	193	Ranging from $0.012m^3$ to $1366.117m^3$<br>Average: $7.153m^3$
	Average space per bird in the cage	176	Ranging from $6768cm^3$ to $1366.117m^3$<br>Average: $7.203m^3$
	Total number of birds in household	218	Ranging from 1 to 36<br>Average: 4.14
Habits	Frequency of socializing with birds from a different household	170	Every day or every other day: 10.59%<br>Once or twice per month: 8.24%<br>Once or twice per half a year: 14.12%<br>Once or twice per year: 12.35%<br>Never: 54.71%
	Quarantine birds when they are sick	172	Yes: 90.12%<br>No: 9.88%

Table S10. Number of PBFDV-positive fecal samples per species from three respective sources. Confidence intervals (CI) were calculated using the Reiczigel method [1].

Common name	Species name	Total no. of samples (%; 95% CI)	Total no. of samples from households	Total no. of samples from pet shops	Total no. of samples from an animal hospital
Parrots					
**Peach-faced lovebird**	*Agapornis roseicollis*	10/116 (8.62%; 0.00-14.88%)	6/85 (7.06%)	4/11 (36.36%)	0/20
**Cockatiel**	*Nymphicus hollandicus*	0/62 (0.00%; 0.00-6.75%)	0/39	0/6	0/17
**Grey Parrot**	*Psittacus erithacus*	4/52 (7.69%; 0.00-17.83%)	0/34	1/2 (50.00%)	3/16 (18.75%)
**Budgerigar**	*Melopsittacus undulatus*	3/29 (10.34%; 0.00-26.33%)	2/13 (15.38%)	1/11 (9.09%)	0/5
**Monk parakeet**	*Myiopsitta monachus*	0/19 (0.00%; 0.00-18.61%)	0/16	0/0	0/3
**Turquoise-fronted amazon**	*Amazona aestiva*	1/17 (5.88%; 0.00-27.99%)	0/0/11	1/1 (100%)	0/5
**Green-thighed parrot**	*Pionites leucogaster*	1/15 (6.67%; 0.00-31.02%)	1/11 (9.09%)	0/0	0/4
**Sun conure**	*Aratinga solstitialis*	1/13 (7.69%; 0.00-34.76%)	0/10	0/0	1/3 (33.33%)
**Eclectus parrot**	*Eclectus roratus*	0/13 (0.00%; 0.00-25.51%)	0/10	0/0	0/3
**Pacific parrotlet**	*Forpus coelestis*	0/12 (0.00%; 0.00-27.17%)	0/6	0/0	0/6
**Green-cheeked parakeet**	*Pyrrhura molinae*	0/11 (0.00; 0.00-29.06%)	0/11	0/0	0/0
**Galah**	*Eolophus roseicapillus*	2/11 (18.18%; 0.31-48.93%)	1/8 (12.50%)	0/1	1/2 (50.00%)
**Hahn's macaw**	*Diopsittaca nobilis*	2/9 (22.22%; 1.89-56.21%)	1/8 (12.50%)	0/0	1/1 (100.00%)
**Sulphur-crested cockatoo**	*Cacatua galerita*	1/8 (12.50%; 0.00-49.27%)	0/6	0/0	1/2 (50.00%)
**Jardine's parrot**	*Poicephalus gulielmi*	0/8 (0.00%; 0.00-36.56)	0/6	0/0	0/2
**Blue and yellow macaw**	*Ara ararauna*	1/8 (12.50%; 0.00-49.27%)	1/5 (20.00%)	0/0	0/3
**Fischer's lovebird**	*Agapornis fischeri*	1/7 (14.29%; 0.00-53.64%)	1/7 (14.29%)	0/0	0/0
**Senegal parrot**	*Poicephalus senegalus*	0/7 (0.00%; 0.00-39.95%)	0/5	0/2	0/0
**Black-headed parrot**	*Pionites melanocephalus*	0/7 (0.00%; 0.00-39.95%)	0/5	0/0	0/2
**Yellow-crowned amazon**	*Amazona ochrocephala*	1/7 (14.29%; 0.00-53.64%)	0/3	1/1 (100.00%)	0/3
**Ring-necked parakeet**	*Psittacula krameri*	0/6 (0.00%; 0.00-43.99%)	0/6	0/0	0/0
**Yellow-crested cockatoo**	*Cacatua sulphurea*	0/6 (0.00%; 0.00-43.99%)	0/5	0/0	0/1
**White cockatoo**	*Cacatua alba*	0/6 (0.00%; 0.00-43.99%)	0/4	0/0	0/2
**Lovebird hybrid**	Hybrid of *A. roseicollis and A. fischeri*	0/4 (0.00%; 0.00-72.50%)	0/4	0/0	0/0
**Rainbow lorikeet**	*Trichoglossus moluccanus*	1/4 (25.00%; 0.00-67.11%)	1/4 (25.00%)	0/0	0/0
**Green-winged macaw**	*Ara chloropterus*	0/4 (0.00%; 0.00-72.50%)	0/2	0/0	0/2
**Alexandrine parakeet**	*Psittacula eupatria*	1/3 (33.33%; 2.52-81.75%)	0/1	0/0	1/2 (50.00%)

**Moustached parakeet**	*Psittacula alexandri*	0/3 (0.00%; 0.00-62.49%)	0/3	0/0	0/0
**Golden collared macaw**	*Primolius auricollis*	0/3 (0.00%; 0.00-62.49%)	0/0	0/2	0/1
**Orange-winged amazon**	*Amazona amazonica*	0/2 (0.00%; 0.00-72.31%)	0/2	0/0	0/0
**Blue-eyed cockatoo**	*Cacatua ophthalmica*	0/2 (0.00%; 0.00-72.31%)	0/2	0/0	0/0
**Ducorps's cockatoo**	*Cacatua ducorpsii*	0/2 (0.00%; 0.00-72.31%)	0/2	0/0	0/0
**Masked lovebird**	*Agapornis personatus*	0/2 (0.00%; 0.00-72.31%)	0/2	0/0	0/0
**Salmon-crested cockatoo**	*Cacatua moluccensis*	0/2 (0.00%; 0.00-72.31%)	0/1	0/0	0/1
**Unclassified Amazon**	*Amazona*	0/2 (0.00%; 0.00-72.31%)	0/0	0/0	0/2
**Little corella**	*Cacatua sanguinea*	0/2 (0.00%; 0.00-72.31%)	0/0	0/0	0/2
**Black-capped Lory**	*Lorius lory*	1/2 (50.00%; 6.71-93.29%)	0/0	1/2 (50.00%)	0/0
**Major Mitchell's cockatoo**	*Lophochroa leadbeateri*	1/1 (100.00%; 14.61-100.00%)	1/1 (100.00%)	0/0	0/0
**Cape parrot**	*Poicephalus robustus*	0/1 (0.00%; 0.00-85.39%)	0/1	0/0	0/0
**Yellow-naped amazon**	*Amazona auropalliata*	0/1 (0.00%; 0.00-85.39%)	0/0	0/0	0/1
**Tanimbar corella**	*Cacatua goffiniana*	0/1 (0.00%; 0.00-85.39%)	0/0	0/0	0/1
**Unclassified Cockatoos**	*Cacatuidea*	0/1 (0.00%; 0.00-85.39%)	0/0	0/0	0/1
**Dusty parrot**	*Pionus fuscus*	1/1 (100.00%; 14.61-100.00%)	0/0	0/0	1/1 (100.00%)
**Total number of samples collected from parrots**		33/492 (6.71%; 0.00-10.02%)	15/339 (4.42%; 0.00-7.89%)	9/39 (23.08%; 8.95-38.30%)	9/114 (7.89%; 0.00-14.09%)
Non-parrot species					
**Oriental magpie-robin**	*Copsychus saularis*	0/6 (0.00%; 0.00-43.99%)	0/0	0/6	0/0
**Swinhoe's White-eye**	*Zosterops simplex*	3/6 (50.00%; 16.54-83.46%)	0/0	3/6 (50.00%)	0/0
**Zebra finch**	*Taeniopygia guttata*	0/4 (0.00%; 0.00-72.50%)	0/4	0/0	0/0
**Java sparrow**	*Padda oryzivora*	0/3 (0.00%; 0.00-62.49%)	0/3	0/0	0/0
**Yellow-fronted canary**	*Crithagra mozambica*	0/2 (0.00%; 0.00-72.31%)	0/0	0/2	0/0
**Common hill myna**	*Gracula religiosa*	1/2 (50.00%; 6.71-93.29%)	0/0	1/1 (100.00%)	0/1
**Common Mynah**	*Acridotheres tristis*	0/1 (0.00%; 0.00-85.39%)	0/0	0/0	0/1
**Total number of samples collected from non-parrot birds**		4/24 (16.67%; 2.17-35.98%)	0/7	4/15 (26.67%; 4.36-65.00%)	0/2
**Total number of samples collected**		37/516 (7.17%; 0.00-10.48%)	15/346 (4.34%; 0.00-7.77%)	13/54 (24.07%; 11.05-36.86%)	9/116 (7.76%; 0.00-13.87%)

Table S11. Number of PBFD-positive birds with and without symptoms in each species (N=36).

Species	With symptoms	Without symptoms
Agapornis roseicollis	0	10
Psittacus erithacus	3 (Feather loss; Feather destructive behaviour; weight loss)	1
Melopsittacus undulatus	0	3
Zosterops simplex	0	3
Diopsittaca nobilis	1 (Feather destructive behaviour; chronic uropygial gland impaction)	1
Eolophus roseicapillus	1 (Feather destructive behaviour)	1
Amazona ochrocephala	1 (Feather loss)	0
Lorius lory	1 (Watery feces)	0
Psittacula eupatria	1 (Feather loss)	0
Ara ararauna	1 (Feather loss)	0
Agapornis fischeri	1 (Lethargy)	0
Cacatua galerita	1 (Lethargy; underweight)	0
Trichoglossus moluccanus	0	1
Gracula religiosa	0	1
Amazona aestiva	0	1
Lophochroa leadbeateri	0	1
Pionites leucogaster	0	1
Aratinga solstitialis	0	1

Table S12 List of BFDV-positive fecal samples obtained from the three different sources. Confidence intervals (CI) were calculated using the Reiczigel method [1]. Only the species with positive samples are listed.

**Common name**	Species name	Total no. of BFDV-positive samples / all samples (Prevalence; 95% CI)	No. of BFDV - positive samples / all samples from **households** (Prevalence)	No. of BFDV - positive samples / all samples from **pet shops** (Prevalence)	No. of BFDV - positive samples / all samples from **an animal hospital** (Prevalence)
**Parrots**					
**Peach-faced lovebird**	*Agapornis roseicollis*	1/116 (0.86%; 0.00-5.55%)	1/85 (1.18%)	0/11	0/20
**Green-thighed parrot**	*Pionites leucogaster*	1/15 (6.67%; 0.00-31.02%)	1/11 (9.09%)	0/0	0/4
**White cockatoo**	*Cacatua alba*	1/6 (16.67%; 0.00-58.81%)	1/4 (25.00%)	0	0/2
**Total no. of BFDV-positive samples from parrots (prevalence; 95% CI)**		3/492 (0.61%; 0.00-3.49%)	3/339 (0.88%; 0.00-4.01%)	0/39	0/114
**Total no. of BFDV-positive samples from all species (prevalence; 95% CI)**		3/516 (0.58%; 0.00-3.43%)	3/346 (0.87%; 0.00-3.98%)	0/54	0/116

Maximum Likelihood (ML) trees of PBFDV *Rep* and *Cap* sequences with outgroups

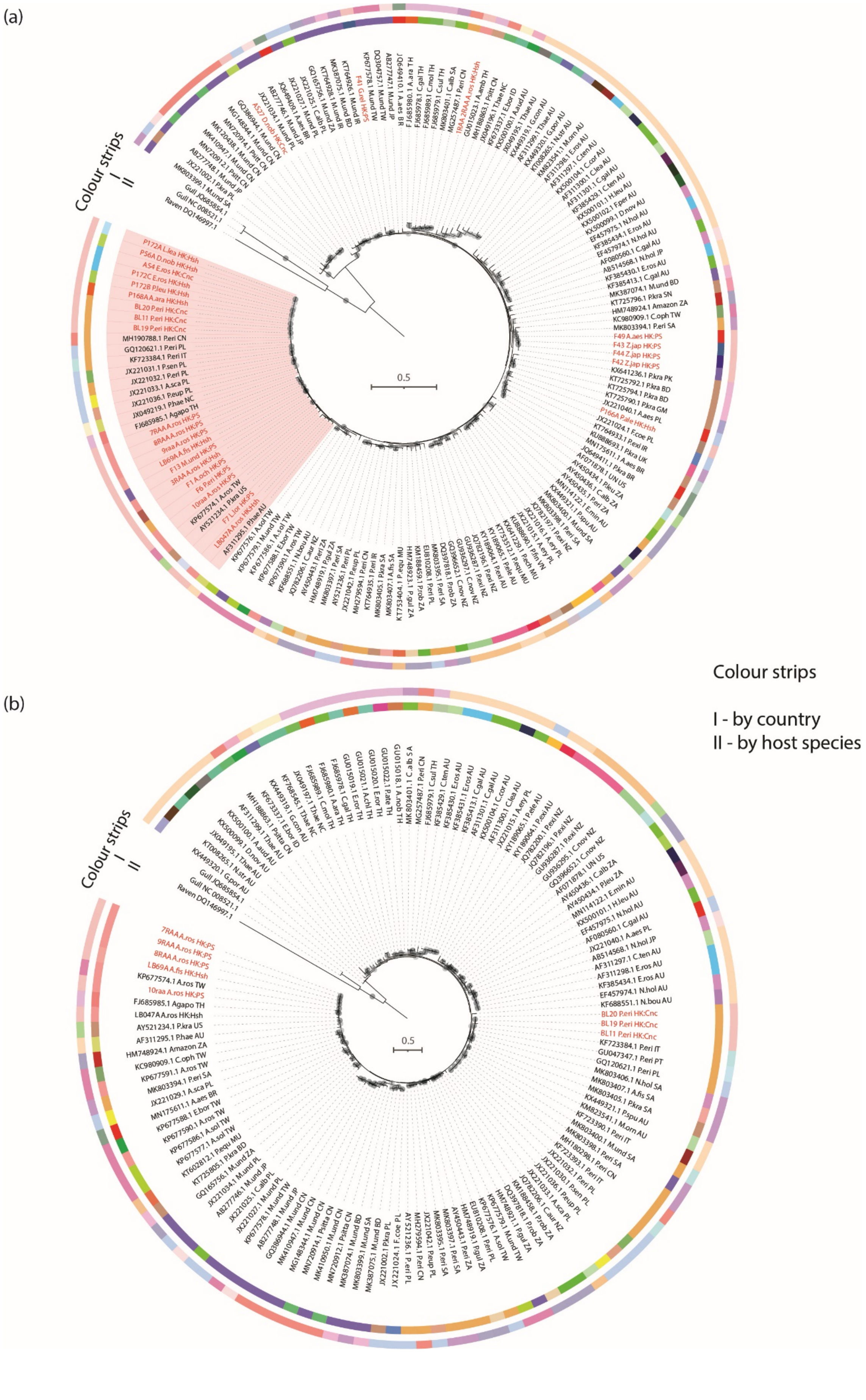

Fig. S1 presents two maximum likelihood (ML) trees constructed using *Rep* and *Cap* gene sequences. (a) The *Rep* tree includes 29 isolates identified in the study and 124 sequences from GenBank. The majority of Hong Kong sequences form a monophyletic group, which is shaded in red. (b) The *Cap* tree includes 9 individuals from Hong Kong and 119 sequences from GenBank. The tree includes three outgroups, which are a raven circovirus sequence (DQ146997.1) and two gull circovirus sequences (JQ685854.1 and NC_008521.1). The outer color strips indicate the country of origin of the isolates, while the inner color strips represent the host species. Bootstrap values are indicated by branch colors. The list of retrieved sequences is available in Table S7 in supplementary material 1.

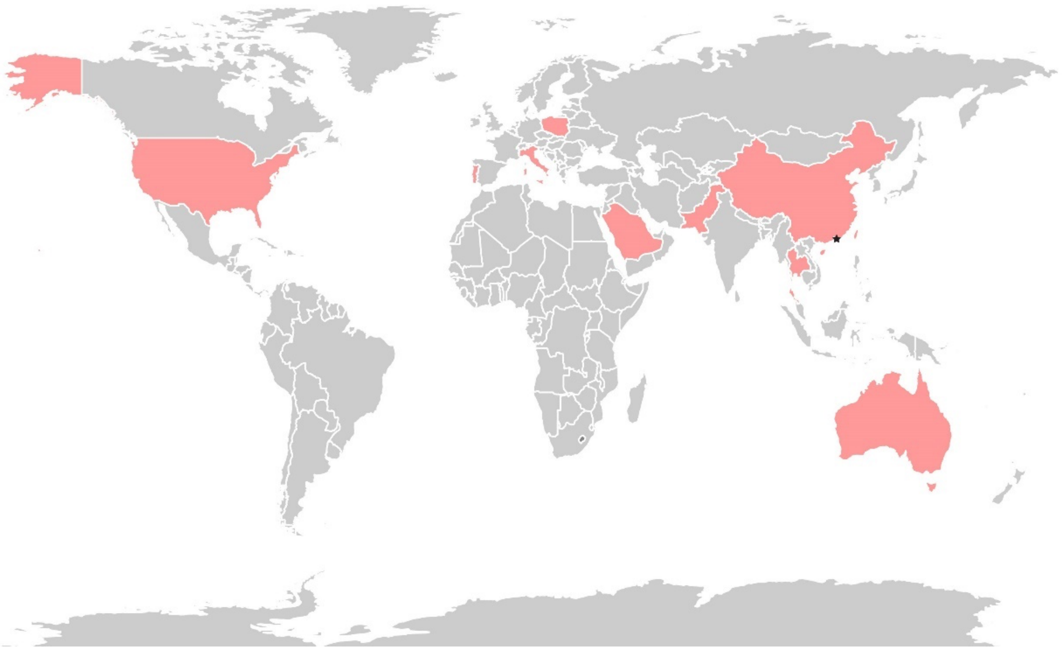

Fig. S2 World map indicating the countries or regions with PBFDV sequences that are closely related to those in Hong Kong. Corresponding countries or regions are highlighted in pink, which include mainland China, Taiwan, Thailand, Pakistan, Saudi Arabia, Italy, Poland, Portugal, New Caledonia, Australia, and USA. Location of Hong Kong is indicated by a star.

MCA results of birds from households only

To conduct the MCA analysis, we considered a variety of variables listed in Table S9, which included bird species, genus, age, as well as the month and season of sampling.

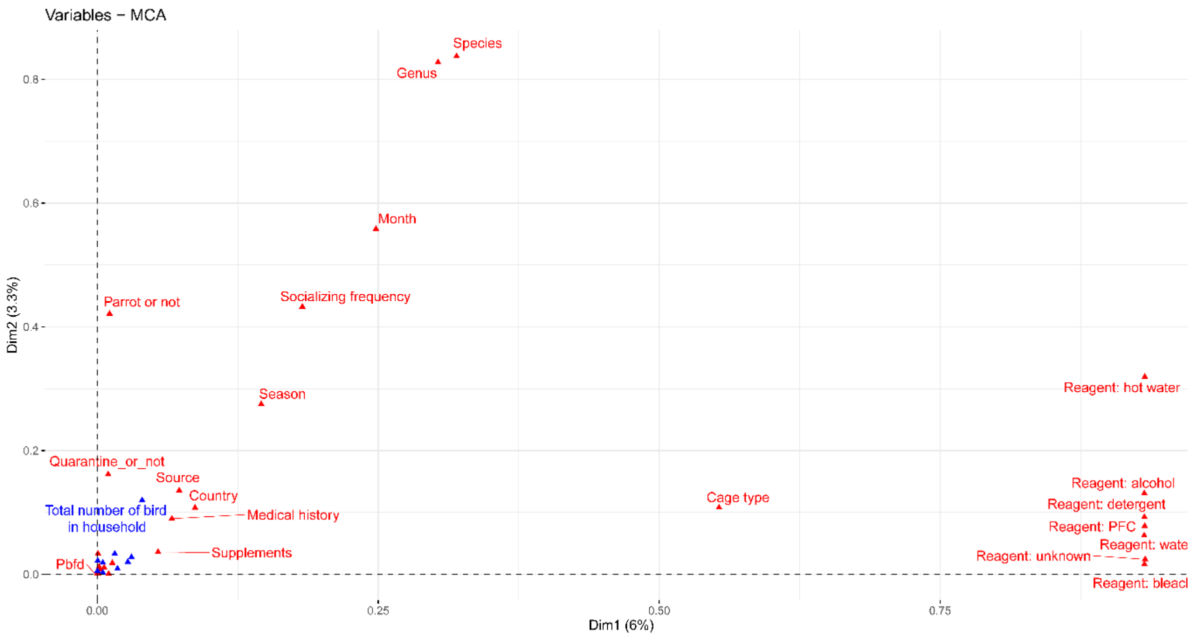

Fig S3 Correspondence plot displaying the relationship between variables in two dimensions. Dimension 1 (Dim1) was best explained by the sources of the birds, while dimension 2 (Dim2) was best explained by the month and season of sampling. The plot is based on data from 221 pet birds in households and includes both qualitative variables (in red) and quantitative variables (in blue).

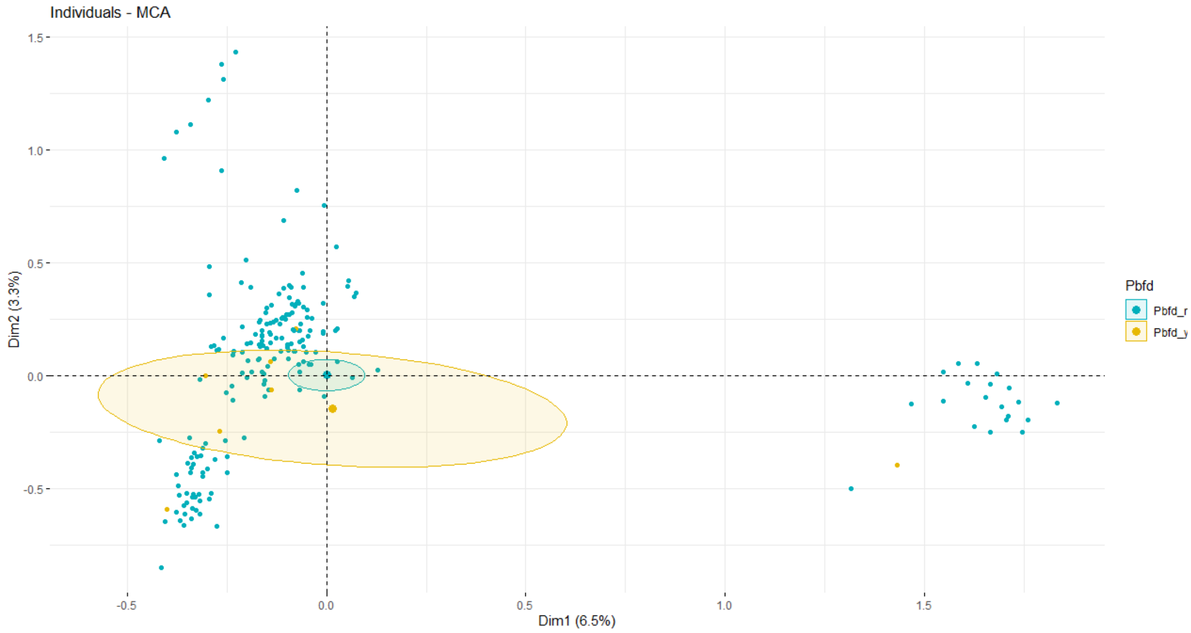

Fig S4 Correspondence plot displaying individual data points in two dimensions, specifically Dimension 1 (Dim1) and Dimension 2 (Dim2), using data from 221 birds from households. Confidence ellipses were drawn around the mean points of PBFDV-negative and PBFDV-positive data points, which are denoted as "Pbfd_n" and "Pbfd_y", respectively.

MCA results of birds from all sources

To conduct the MCA analysis, we considered a variety of variables listed in Table S9, which included bird species, genus, age, as well as the month and season of sampling.

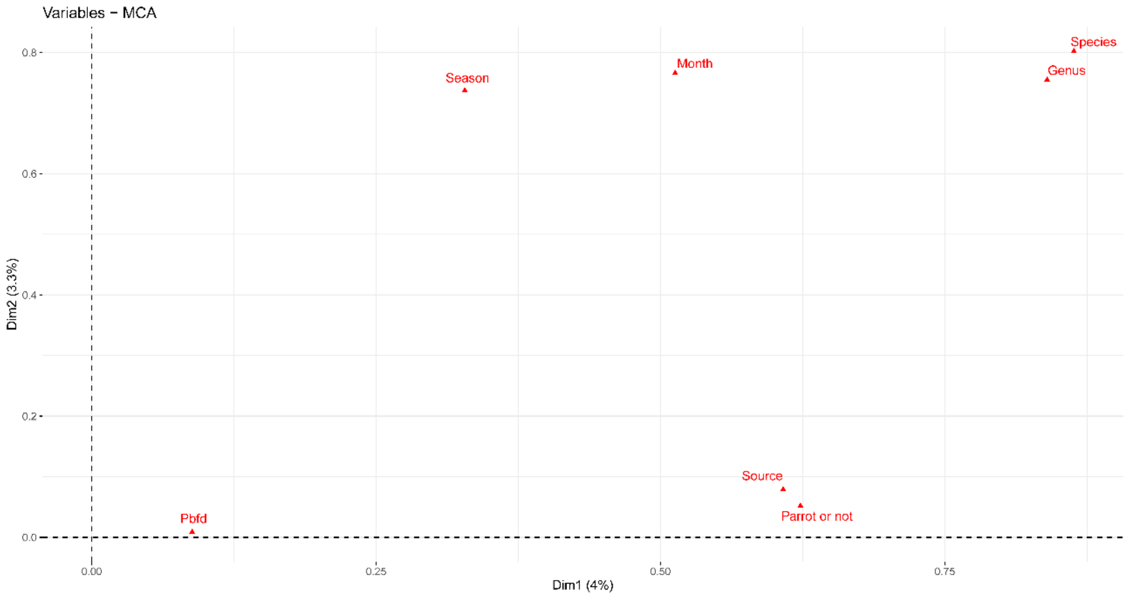

Fig S5 Correspondence plot displaying the relationship between variables in two dimensions. Dimension 1 (Dim1) was best explained by the sources of the birds, while dimension 2 (Dim2) best explained by the month and season of sampling. The plot was based on data from 221 pet birds from households, as well as all birds sampled from pet shops, and animal clinic.

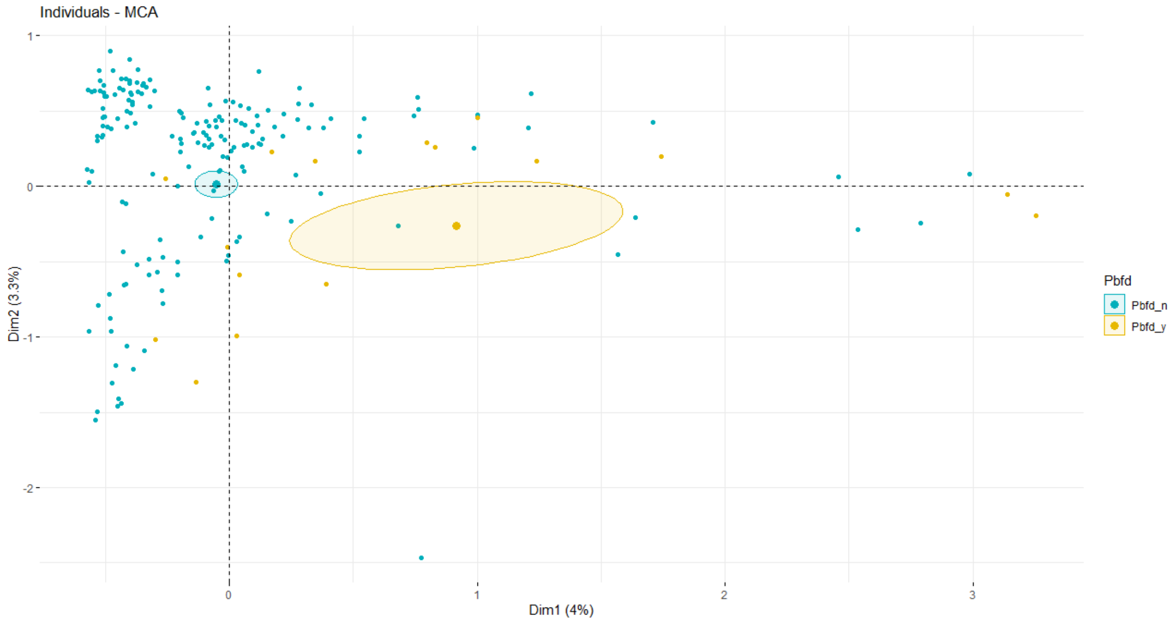

Fig. S6 Correspondence plot displaying individual data points in two dimensions, specifically Dimension 1 (Dim1) and Dimension 2 (Dim2). The plot was based on data from 221 pet birds from households, as well as all birds sampled from pet shops and animal clinic. Confidence ellipses were drawn around the mean points of PBFDV-negative and PBFDV-positive data points.

References

(1) Lang Z, & Reiczigel J (2014) Confidence limits for prevalence of disease adjusted for estimated sensitivity and specificity. Prev Vet Med 113:13-22. https://doi.org/10.1016/j.prevetmed.2013.09.015
